# Supplementary material for: Ultimate Photo-Thermo-Acoustic Efficiency of Graphene Aerogels
Source: Sci Rep. 2019 Sep 16;9:13386. doi: 10.1038/s41598-019-50082-7 (PMC6746718; doi:10.1038/s41598-019-50082-7)
Supplement: Supplementary file 1 — SUPPLEMENTARY INFO [file 41598_2019_50082_MOESM1_ESM.pdf]

Supplementary Information to Ultimate Photo-Thermo-Acoustic Efficiency of Graphene Aerogels

Francesco De Nicola,[1,*] Lorenzo Donato Tenuzzo,[2] Ilenia Viola,[3] Rujing Zhang,[4] Hongwei Zhu,[4] Augusto Marcelli,[5,6] and Stefano Lupi[2,1]

[1] *Graphene Labs, Istituto Italiano di Tecnologia, Via Morego 30, 16163 Genova, Italy*

[2] *Department of Physics, University of Rome La Sapienza, P.le A. Moro 5, 00185 Rome, Italy*

[3] *CNR NANOTEC-Institute of Nanotechnology, S.Li.M Lab, Department of Physics, University of Rome La Sapienza, P.le A. Moro 5, 00185 Rome, Italy*

[4] *State Key Laboratory of New Ceramics and Fine Processing, School of Materials Science and Engineering, Tsinghua University, Beijing 100084, China*

[5] *INFN-Laboratori Nazionali di Frascati, Via Enrico Fermi 40, 00044 Frascati (RM), Italy*

[6] *RICMASS, Rome International Center for Materials Science Superstripes, Via dei Sabelli 119A, 00185 Rome, Italy*

1. PHOTO-THERMO-ACOUSTIC MODEL

A general analytical solution for the root-mean-square sound pressure amplitude in the photo-thermo-acoustic (PTA) model can be derived by the thermo-acoustic model[1,2] as follows

$$p_{rms} = \frac{R_0}{\sqrt{2}r_0}\frac{\gamma - 1}{v_g}\frac{e_g}{M(f)e_s + e_g}q_0(\lambda)\mathcal{D}(\theta, \phi), \quad (1)$$

with R_0/r_0 the Rayleigh correction for far-field regime, being r_0 the distance from the sound source and $R_0 = S/\lambda_g$ with $\lambda_g = v_g/f$ the gas acoustic wavelength, S the sample illuminated surface area, and v_g and f the gas speed and frequency of sound, respectively; $e_i = \sqrt{k_i\rho_i C_{p,i}}$ the thermal effusivity of the sample ($i = s$) and the gas ($i = g$), with k_i the thermal conductivity, ρ_i the mass density, and $C_{p,i}$ the specific heat capacity; $\gamma = C_{p,g}/C_{v,g}$ the gas adiabatic index, $q_0(\lambda) = Q_0(\lambda)/S$ the power density of the light of wavelength λ absorbed by the sample with power amplitude $Q_0(\lambda) = S\left[a(\lambda)\sigma\left(T_1^4 - T_2^4\right) - h\left(T_1 - T_2\right)\right]$, with $a(\lambda) = 1 - R(\lambda)$ the sample absorptivity and $R(\lambda)$ the sample reflectivity, σ the Stephen-Boltzmann constant, $T_{1,2}$ the light source and sample temperature, respectively, and h the convection coefficient; $\mathcal{D}(\theta, \phi) = \mathrm{sinc}[\frac{1}{2}\kappa L_x \sin(\theta)\cos(\phi)]\mathrm{sinc}[\frac{1}{2}\kappa L_y \sin(\theta)\sin(\phi)]$ the far-field directivity, with $\kappa = 2\pi/\lambda_g$ the sound wavevector, $L_{x,y}$ the lateral dimensions of the illuminated spot (i.e., the PTA diaphragm), and θ and ϕ the azimuthal and polar angle, respectively. For a self-standing sample with no backing material[2] $M(f) = k_s\sigma_s \tanh(\sigma_s L_s)$, where L_s is the sample thickness and $\sigma_s = \sqrt{i2\pi f/\alpha_s}$, with α_s the thermal diffusivity of the sample. Equation 1 practically reduces to

$$p_{rms} = \frac{fQ_0(\lambda)}{\sqrt{2}r_0 C_{p,g}T_g}\frac{e_g}{M(f)e_s + e_g}\mathcal{D}(\theta, \phi), \quad (2)$$

with T_g the gas temperature. Eq. 2 holds at low frequency when the sample thickness $L_s < 2\pi\mu$, being $\mu = \sqrt{\alpha_s/\pi f}$ the thermal diffusion length. On the other hand, at high frequency when $L_s > 2\pi\mu$, $M(f) = 1$ and Eq. 2 reads

$$p_{rms} = \frac{fQ_0(\lambda)}{\sqrt{2}r_0 C_{p,g}T_g}\frac{e_g}{e_s + e_g}\mathcal{D}(\theta, \phi). \quad (3)$$

This is our case, as for graphene aerogels $\mu = 10$ μm-0.1 mm and $d = 2 - 20$ mm. From the above equation is clear that $e_s > -e_g$, therefore $\rho_s \geq \rho_0 \equiv e_g^2/k_s C_{p,s}$. The ultimate limit of the PTA model is for $\mathcal{D}(\theta, \phi) = 1$ and $e_s \to e_g$ or $\rho \to \rho_0$, which reads

$$p_{rms} = \frac{fQ_0(\lambda)}{2\sqrt{2}r_0 C_{p,g}T_g}. \quad (4)$$

This means that for a given medium, light power, and distance from the source, the sound pressure is only frequency limited. Since the corresponding sound intensity is $I = p_{rms}^2/\rho_g v_g$, the acoustic power in half space $P_{ac} = Ir^2 \int_0^{2\pi} \int_0^{\pi/2} \mathcal{D}^2(\theta,\phi) \sin\theta d\theta d\phi$ has a maximum at distance $r = r_0$ for a point-source, which reads

$$P_{ac} = \pi \frac{f^2 Q_0^2(\lambda)}{4\rho_g v_g C_{p,g}^2 T_g^2} \tag{5}$$

and for a directive source

$$P_{ac} = \frac{Q_0^2(\lambda) v_g}{4\pi L_x^2 \rho_g C_{p,g}^2 T_g^2}, \tag{6}$$

being $f = v_g/\pi L_x$ the high-frequency cut-off of the acoustic power. Therefore, the ultimate PTA efficiency $\eta = P_{ac}/Q_0(\lambda)$ is

$$\eta = \pi \frac{f^2 Q_0(\lambda)}{4\rho_g v_g C_{p,g}^2 T_g^2}, \tag{7}$$

and beyond the audible range, where the source becomes directive reads

$$\eta = \frac{q_0(\lambda) v_g}{8\rho_g C_{p,g}^2 T_g^2}. \tag{8}$$

2. MASS DENSITY CHARACTERIZATION

The mass density of the graphene aerogels can be estimated at first approximation by $\rho = m/V$. However, this is the total mass density, as aerogels are matter with a mix phase of gas and solid. Therefore, a better estimation of the solid mass density can be provided by the effective medium approximation $\rho = \rho_c(1 - \Phi_{air}) + \rho_{air}\Phi_{air}$, being $\Phi_c + \Phi_{air} = 1$, where ρ_c and $\Phi_c = V_c/V$ are respectively the density and the volume fraction of the carbon phase, while $\rho_{air} = 1.225$ kg/m^3 and $\Phi_{air} = V_{air}/V$ are the density and the volume fraction of air, respectively. Hence, the effective mass density of the sample is defined as $\rho_s = \rho_c(1 - \Phi_{air})$.

In order to obtain the carbon mass density ρ_c, we estimated the air volume fraction by carrying out contact angle measurements, being the aerogels hydrophobic in the Cassie-Baxter regime[3] (Figure S1). Images of sessile water drops cast on over 30 graphene aerogels were acquired by Dataphysics OCA instrument and analyzed by its software. In order to estimate the average, maximum, and minimum contact angle on the heterogeneous and porous aerogel surface (contact angle hysteresis $\approx 15°$), static, advanced, and receding contact angles[3] were measured, respectively, by increasing and decreasing the volume of the water drop by

1 μL step. The deionized water (18 MΩcm) drop volume used to achieve the static contact angles of the samples was $V = 15$ μL. Moreover, every contact angle was measured 15 s after drop casting, to ensure that the droplet reached its equilibrium position.

The Cassie-Baxter law in the hydrophobic regime reads[3]

$$\cos\Theta^* = (1 - \Phi_{air})\cos\Theta_Y - \Phi_{air}, \tag{9}$$

where Φ is the volume fraction of air pockets underneath the water droplet, Θ^* is the measured apparent contact angle, and $\Theta_Y = 87°$ the corresponding Young contact angle measured on a highly oriented pyrolytic graphite (HOPG) substrate. Since the air pockets occur due to the aerogel porous surface, we estimated an aerogel air volume fraction $\Phi_{air} = 0.43 - 0.92$ by considering the air pocket volume fraction underneath the water droplets cast on several spots of the aerogel surface. Since the aerogel is a three-dimensional fractal solid with a self-similar structure, we assumed that the air volume fraction on the aerogel surface is the same in the aerogel volume.

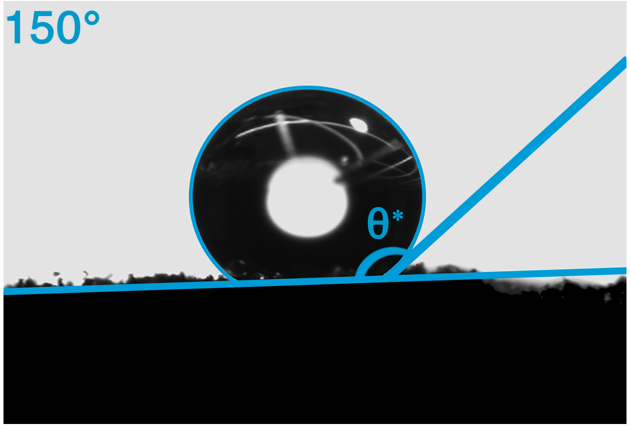

FIG. 1. Image of a water droplet cast on a super-hydrophobic ($\Theta^* = 150 \pm 15°$) graphene aerogel with air volume fraction $\Phi_{air} = 0.87$. The error on the static contact angle is estimated by the contact angle hysteresis.

3. OPTICAL CHARACTERIZATION

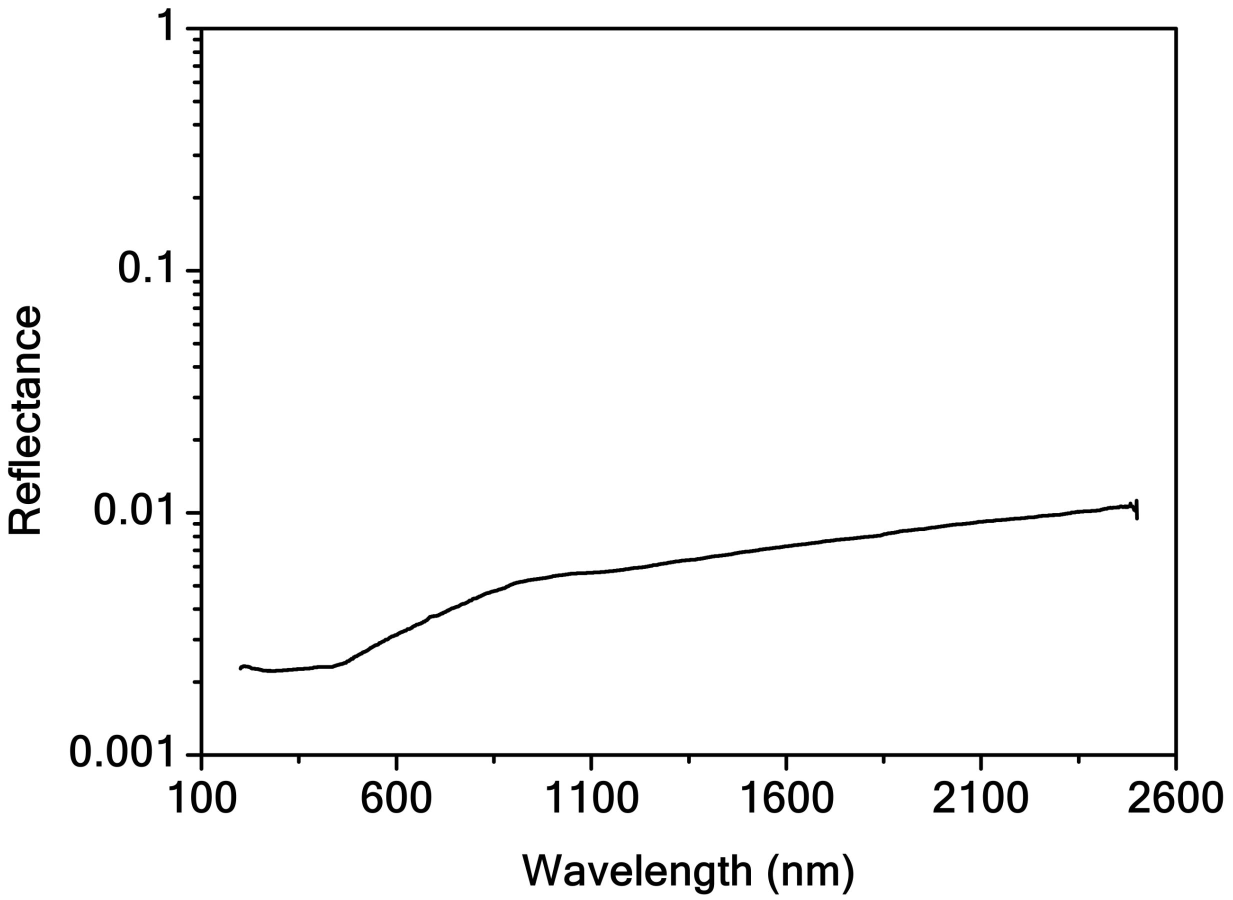

FIG. 2. Optical reflectance spectrum of a graphene aerogel.

4. THERMAL CHARACTERIZATION

The graphene aerogel thermal properties were investigated by a Fluke Ti20 thermal camera. The sample base surface were illuminated by a panchromatic LED light source of power $Q_1 = 0.5$ W with a Gaussian spot profile (Figure S3a, b). Heat is exchanged in time between the LED and the sample by radiation and convection, which reads

$$Q_0(\lambda) = S\left[a(\lambda)\sigma\left(T_1^4 - T_2^4\right) - h\left(T_1 - T_2\right)\right], \tag{10}$$

with $S \approx 2$ cm^2 and $a(\lambda) \equiv 1 - R(\lambda) \approx 0.99$ the sample illuminated surface area and absorptivity, respectively, $R(\lambda) < 0.01$ the sample reflectivity, σ the Stephen-Boltzmann

constant, $T_{1,2}$ the light source and sample temperature, respectively, and $h = 1 - 10$ W/m^{2}K the experimental air convection coefficient, which is compatible with the value reported for air laminar flow[4]. In our experiments, we observed that the heat convection term is negligible and all the light power is absorbed by the sample ($Q_0 = Q_1$).

The heat-up phase was recorded in order to obtain the heat capacity $C_s \approx 10^{-2} \pm 10^{-3}$ J/K of the samples (Figure S3c). Since the Debye temperature of carbon allotropes is $\Theta_D > 1000$ K[5], the heat capacity is not stationary in the range of temperature considered. Therefore, since the steady state is the one that contributes to the sound generation, we took into account the maximum value of heat capacity

$$C_s(t) = Q_0(\lambda)\frac{t - t_0}{T_{MAX} - T_{MIN}}, \tag{11}$$

where t_0 is the initial time and $T_{MAX,MIN}$ the sample maximum and minimum temperature, respectively.

Thermal conductivity $k_s \approx 10^{-2} \pm 10^{-3}$ W/mK of the samples was derived by a thermal profile image of the illuminated sample base surface at the steady state (Figure S3d), by fitting data with the Fourier law of heat conduction in cylindrical coordinates

$$k_s = \frac{Q_0(\lambda) ln(\frac{r_2}{r_1})}{2\pi d \Delta T}, \tag{12}$$

where $r_{1,2}$ are the coordinates of the radial thermal profile of temperature T and $d \approx 1.5$ cm is the sample thickness.

Thermal diffusivity α_s of the samples was studied by illuminating the samples on the base surface and recording the heat-up phase at the orthogonal lateral surface (Figure S3e). Data was fit by the law[6]

$$T(t) = T_{MAX}\left[1 + 2\sum_{n=1}^{\infty}(-1)^n\, exp\left(\frac{-n^2\pi^2\alpha_s t}{d^2}\right)\right], \tag{13}$$

in order to achieve the value of $\alpha_s \approx 10^{-6} \pm 10^{-7}$ m^2/s.

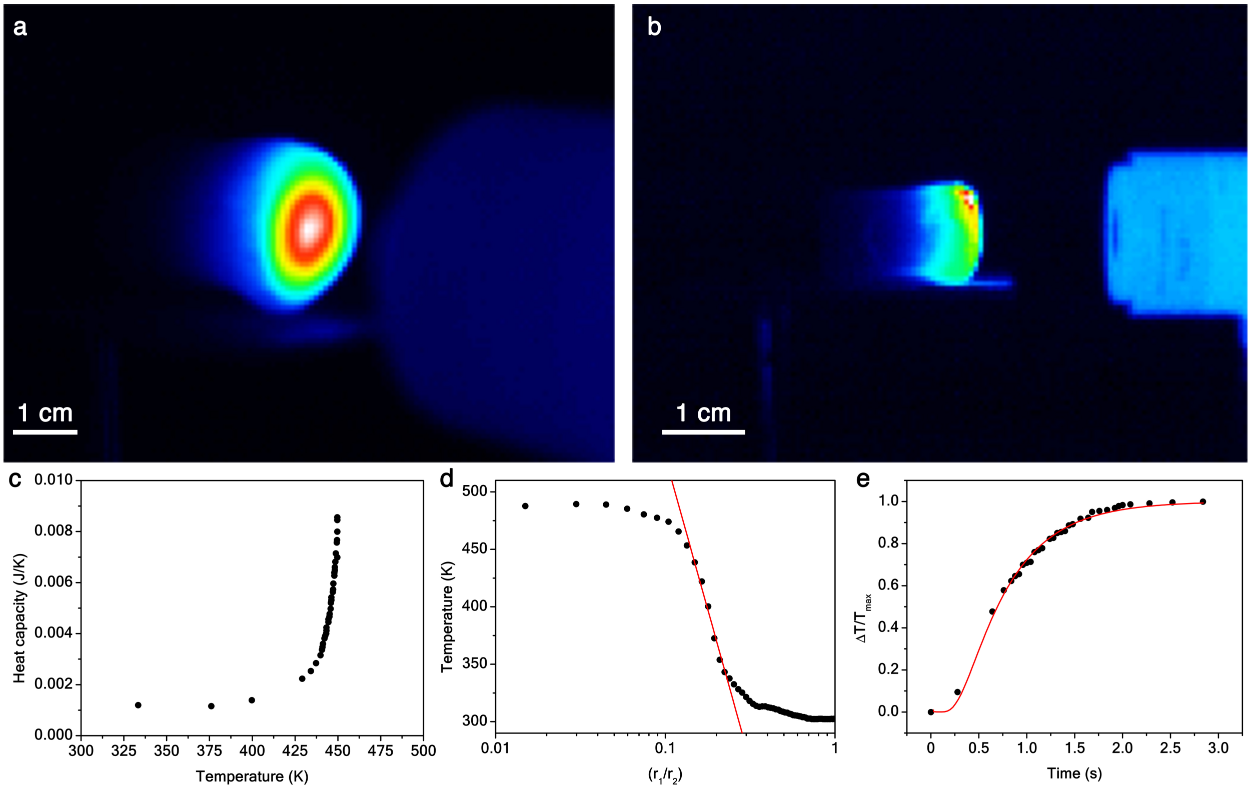

FIG. 3. **a**, Thermal profile image of the illuminated sample base surface. **b**, Thermal profile image of the illuminated sample lateral surface. **c**, Heat capacity of a graphene aerogel as a function of temperature. **d**, Radial thermal profile of the graphene aerogel illuminated surface as a function of the relative coordinate. Data is fit by the Fourier law in cylindrical coordinates (red solid line). **e**, Temperature rise as a function of time at 4 mm from the graphene aerogel illuminated surface. Data is fit by the diffusivity law (red solid line).

5. ACOUSTIC CHARACTERIZATION OF COMMERCIAL LOUDSPEAKERS

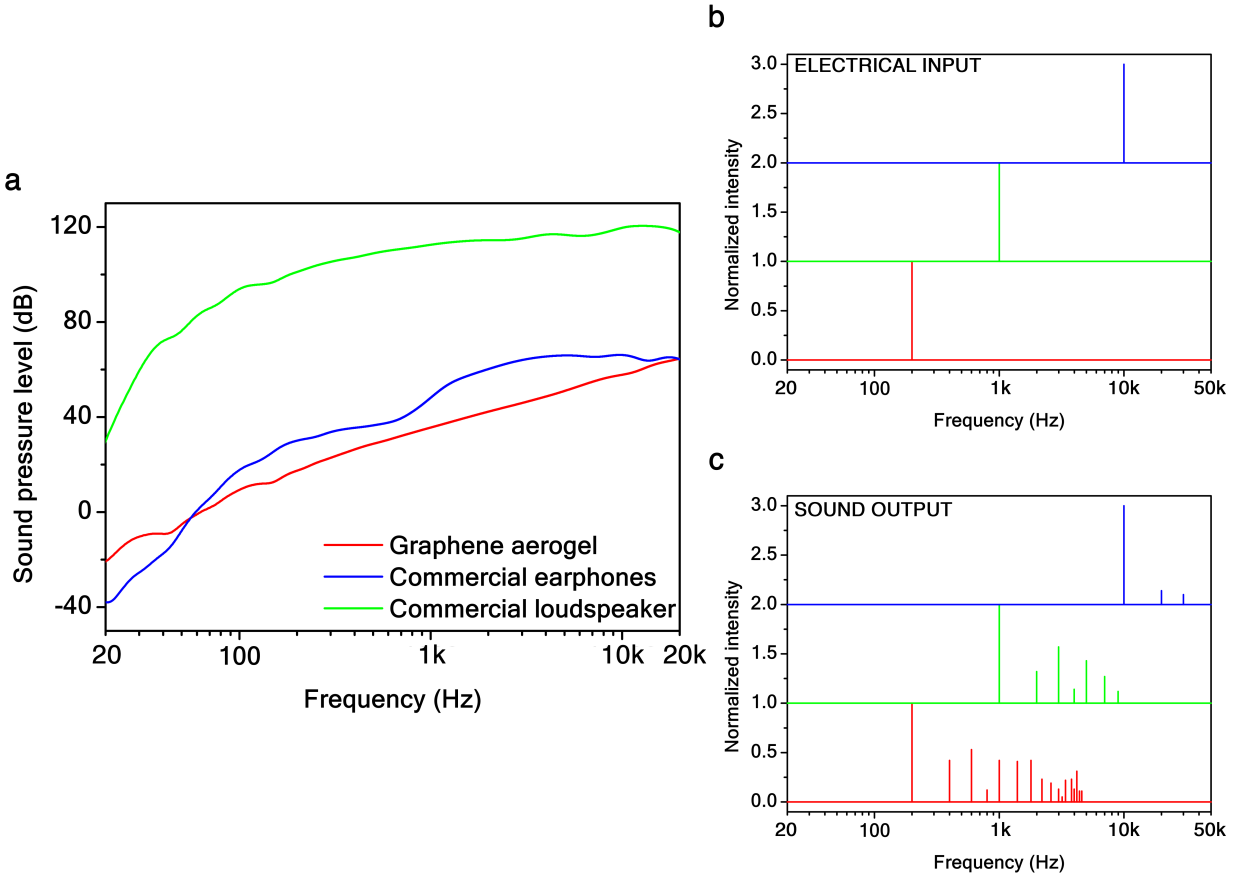

FIG. 4. **a**, Unweighted SPL frequency response at the input power of 1 W and recorded at 1 m distance from the source for a graphene aerogel with effective mass density ρ_s =0.25 kg/m^3 (red solid curve), commercial Apple earphones (blue solid curve), and commercial Soundvision loudspeakers (green solid curve). Fast Fourier transform of the electrical input (**b**) and sound output (**c**) signals at 200 Hz, 1 kHz, and 10 kHz for the commercial Soundvision loudspeakers. The input signal is undistorted, while the output signal has THD(200 Hz)= 1%, THD(1 kHz)= 0.8%, and THD(10 kHz)= 0.2%.

Sample	p_{rms} (mPa)	L_p (dB)	L_W (dB)	η	Sensitivity (dB)
Soundvision loudspeakers	7963	112	103	$2 \cdot 10^{-2}$	-8
Apple earphones	5	48	56	$4 \cdot 10^{-7}$	-72
Freestanding G aerogel (this work)	1.30	36	44	$3 \cdot 10^{-8}$	-84
Freestanding CNT film[7]	1.20	35	43	$2 \cdot 10^{-8}$	-85
MLG on paper[8]	0.72	31	39	$8 \cdot 10^{-9}$	-89
Freestanding Al film[1]	0.65	30	38	$7 \cdot 10^{-9}$	-90
Freestanding G Aerogel[7]	0.50	28	36	$4 \cdot 10^{-9}$	-92
Freestanding n-G aerogel[9]	0.42	26	34	$3 \cdot 10^{-9}$	-93
MLG on polymer[10]	0.30	23	31	$1 \cdot 10^{-9}$	-96
Freestanding G aerogel[11]	0.18	19	27	$5 \cdot 10^{-10}$	-101
Au NWs on glass[12]	0.10	14	22	$2 \cdot 10^{-10}$	-106
Freestanding CNT aerogel[7]	0.06	9	17	$6 \cdot 10^{-11}$	-110
SLG on PDMS[13]	0.06	9	17	$6 \cdot 10^{-11}$	-110
ITO on PET[14]	0.03	3	11	$2 \cdot 10^{-11}$	-117
PEDOT:PSS on glass[15]	0.02	0	8	$6 \cdot 10^{-12}$	-120
Al on porous Si[16]	0.02	0	8	$6 \cdot 10^{-12}$	-120
SLG on Si/SiO_2[17]	0.02	0	8	$6 \cdot 10^{-12}$	-120

TABLE I. Summary of the figures of merit for PTA and thermo-acoustic loudspeakers normalized at 1 W/1 m/1 kHz in half space free-field.

6. EFFICIENCY VS. MASS DENSITY

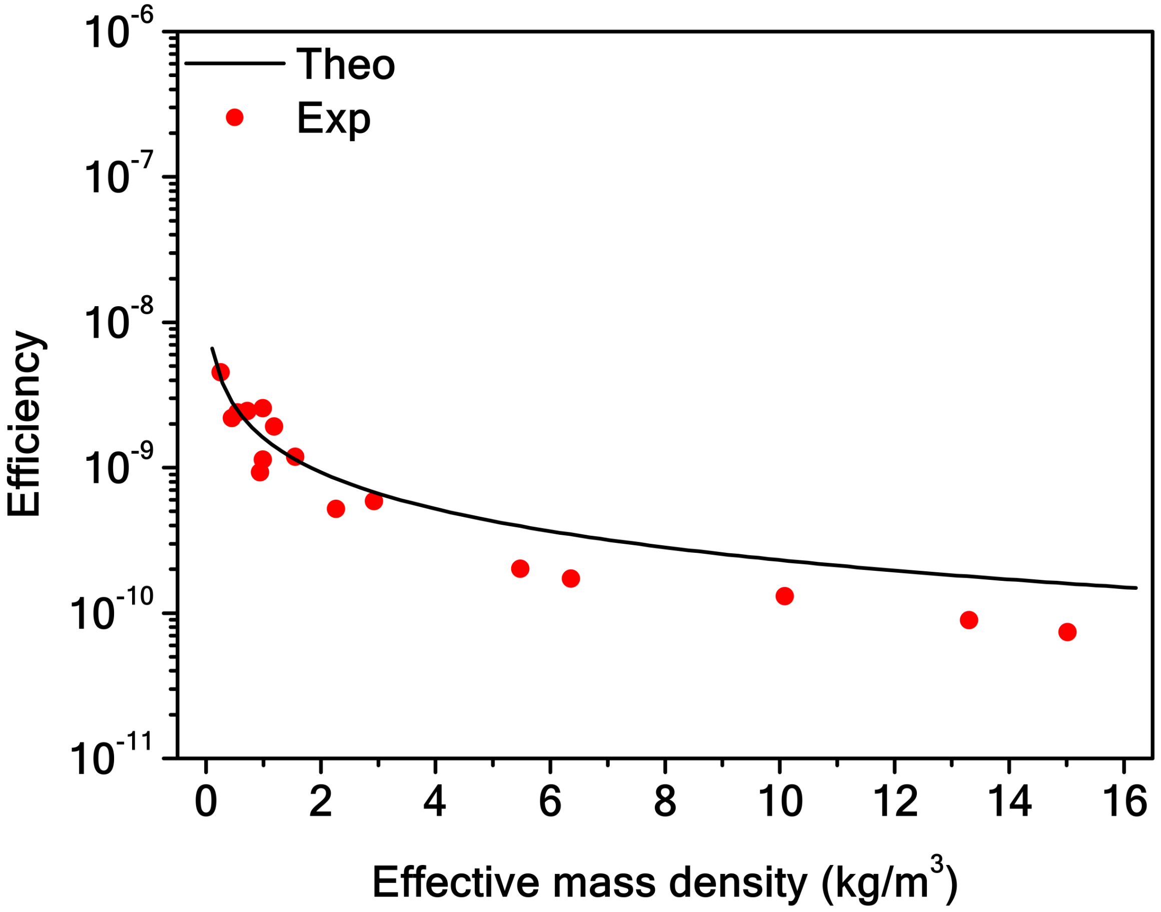

FIG. 5. Unweighted efficiency at 1 W/1 m/1 kHz as a function of the graphene aerogel mass density (red dots). The black solid curve represents the PTA model.

* E-mail: francesco.denicola@iit.it

[1] Vesterinen, V., Niskanen, A. O., Hassel, J. & Helistö, P. Fundamental efficiency of nanothermophones: Modeling and experiments. *Nano Lett.* **10**, 5020–5024 (2010).

[2] Hu, H., Zhu, T. & Xu, J. Model for thermoacoustic emission from solids. *Appl. Phys. Lett.* **96**, 2141011–2141013 (2010).

[3] De Gennes, P.-G., Brochard-Wyart, F. & Quéré, D. *Capillarity and wetting phenomena* (Springer, New York, 2003).

[4] Callen, H. B. *Thermodynamics and an Introduction to Thermostatistics* (John Wiley & Sons, New York, 1985).

[5] Pop, E., Varshney, V. & Roy, A. K. Thermal properties of graphene: Fundamentals and applications. *MRS Bulletin* **37**, 1273–1281 (2012).

[6] Parker, W. J., Jenkins, R. J., Butler, C. P. & Abbott, G. L. Flash method of determining thermal diffusivity, heat capacity, and thermal conductivity. *J. Appl. Phys.* **32**, 1679–1684 (1961).

[7] Aliev, A. E. et al. Alternative nanostructures for thermophones. *ACS Nano* **9**, 4743–4756 (2015).

[8] Tian, H. et al. Graphene-on-paper sound source devices. *ACS Nano* **5**, 4878–4885 (2011).

[9] Kim, C. S. et al. Application of n-doped three-dimensional reduced graphene oxide aerogel to thin film loudspeaker. *ACS Appl. Mater. Interfaces* **8**, 2229522300 (2016).

[10] Kim, C. S. et al. Free-standing graphene thermophone on a polymer-mesh substrate. *Small* **12**, 185–189 (2016).

[11] Fei, W., Zhou, J. & Guo, W. Low-voltage driven graphene foam thermoacoustic speaker. *Small* **11**, 2252–2256 (2015).

[12] Dutta, R. et al. Gold nanowire thermophones. *J. Phys. Chem. C* **118**, 29101–29107 (2014).

[13] Suk, J. W., Kirk, K., Hao, Y., Hall, N. A. & Ruoff, R. S. Thermoacoustic sound generation from monolayer graphene for transparent and flexible sound sources. *Adv. Mater.* **24**, 6342–6347 (2012).

[14] Tian, H. et al. Transparent, flexible, ultrathin sound source devices using indium tin oxide films. *Appl. Phys. Lett.* **99**, 0435031–0435033 (2011).

[15] Tian, H. et al. Poly(3,4-ethylenedioxythiophene):poly(styrenesulfonate)-based organic, ultrathin, and transparent sound-emitting device. *Appl. Phys. Lett.* **99**, 233503 (2011).

[16] Shinoda, H., Nakajima, T., Ueno, K. & Koshida, N. Thermally induced ultrasonic emission from porous silicon. *Nature* **400**, 853–855 (1999).

[17] Heath, M. S. & Horsell, D. W. Multi-frequency sound production and mixing in graphene. *Sci. Rep.* **7**, 13631–13639 (2017).
